# The Evolution of Symbolic Thought: At the Intersection of Schizophrenia Psychopathology, Ethnoarchaeology, and Neuroscience

**DOI:** 10.1007/s11013-024-09873-5

**Published:** 2024-07-12

**Authors:** Matteo Tonna

**Affiliations:** 1 Department of Medicine and Surgery, Psychiatric Unit, University of Parma, Ospedale Maggiore, Padiglione Braga, Viale A. Gramsci 14, 43126 Parma, Italy; 2 Department of Mental Health, Local Health Service, Parma, Italy

**Keywords:** Culture, Development, Embodiment, Language, Self, Sensorimotor

## Abstract

The human capacity for symbolic representation arises, evolutionarily and developmentally, from the exploitation of a widespread sensorimotor network, along a fundamental continuity between embodied and symbolic modes of experience. In this regard, the fine balancing between constrained sensorimotor connections (responsible for self-embodiment processing) and more untethered neural associations (responsible for abstract and symbolic processing) is context dependent and plastically neuromodulated, thus intersubjectively constructed within a specific socio-cultural milieu. Instead, in the schizophrenia spectrum this system falls off catastrophically, due to an unbalance toward too unconstrained sensorimotor connectivity, leading to a profound distortion of self/world relation with a symbolic activity detached from its embodied ground. For this very reason, however, schizophrenia psychopathology may contribute to unveil, in a distorted or magnified way, ubiquitous structural features of human symbolic activity, beneath the various, historically determined cultural systems. In this respect, a comparative approach, linking psychopathology and ethnoarchaeology, allows highlight the following invariant formal characteristics of symbolic processing: (1) Emergence of salient perceptive fragments, which stand out from the perceptual field. (2) Spreading of a multiplicity of new significances with suspension of common-sense meaning. (3) Dynamic and passive character through which meaning proliferation is experienced. This study emphasizes the importance of fine-grained psychopathology to elucidate, within a cross-disciplinary framework, the evolutionarily and developmental pathways that shape the basic structures of human symbolization.

## Introduction

The emergence of symbolic thought represents a tipping-point in the evolution of human mind, marking the split from our ancestors (Bentley & O’Brien, 2012). Growing evidence suggests that this uniquely human capacity, instead of being “encapsulated” in newly acquired cognitive modules, is actually firmly grounded in our bodily enactment, along a continuum from embodied to symbolic mode of experience (Borghi et al., 2022). This continuity lies in fact on the high flexibility of sensorimotor networks, which are plastically re-adapted from rigid neuro-chemical constraints (for concrete representations) to relatively unconstrained connections (to convey more abstract and symbolic concepts) (Mazzuca et al., 2021). In particular, the disproportionate expansion of the cortical mantle during human evolution leads to neural patterning partly freed from chemical signaling gradients, opening up more flexible and dynamic neural configurations, which are more developmentally constructed than phylogenetically fixed (Buckner & Krienen, 2013). The increasing degree of neural “relaxation” paved the way for the extensive exploitation of sensorimotor grounding for symbolic activity, driven by complex bio-cultural feedbacks within specific culturally constructed niches (Stout & Hecht, 2017; Tattersall, 2016).

The importance of cultural developmental forces in shaping human symbolism has been particularly emphasized in ethnoarchaeological accounts. Ethnoarchaeology is an interdisciplinary approach aimed at investigating the relationship of material culture to culture as a whole, both in the living context and as it enters the archaeological record (Nicholas & Kramer, 2001). Under this perspective in fact, human symbolic activity presents a shared, constructed, inter-dependent character, unfolding within a subject–object circular relationship in a given socio-cultural milieu (Hodder, 2011; Malafouris, 2019).

From a psychopathological perspective, schizophrenia is the clinical condition that most dramatically questions the human attitude to symbolization. Patients with schizophrenia in fact typically appear immersed in empty symbolic constructions, while being detached from the common-sense world of shared, everyday meanings (de Vries et al., 2013; Sass, 1992a, b). Remarkably, schizophrenia is a uniquely human condition (Insel, 2010) and, like symbolic activity, it has been specifically linked to the evolution of language (DeLisi, 2021).

Therefore, the present contribution, adopting a cross-disciplinary approach, sought to (1) trace the evolutionary scaffolding of human symbolic activity; (2) compare modes of symbolic representation as manifested in ethnoarchaeological accounts and as reported in schizophrenia psychopathology, with the aim of investigating possible invariant formal features underlying symbolic thought, based on common evolutionary pathways.

## The Inter-dependent Nature of Human Symbolic Evolution

According to an embodied, situated approach, the basic structures of subjectivity develop from the very beginning *within* and *through* a diffuse network of other subjects and worldly objects, mediated by bodily enactment (Gallagher, 2017; Gallese & Ferri, 2014; Laland, 2017). In this connection, also our immediate and meaningful attachment to the world (the *common sense*) spreads from a dynamic engagement with things and bodily manipulation (Chemero, 2019; Park & Baxter, 2022). On the other hand, things, instead of being inert perceptual objects of a detached mind, actually represent a meaningful background of culturally transmitted practices, uses, and affordances, which actively contribute to shape our grasp with the world.

**Fig. 1:**
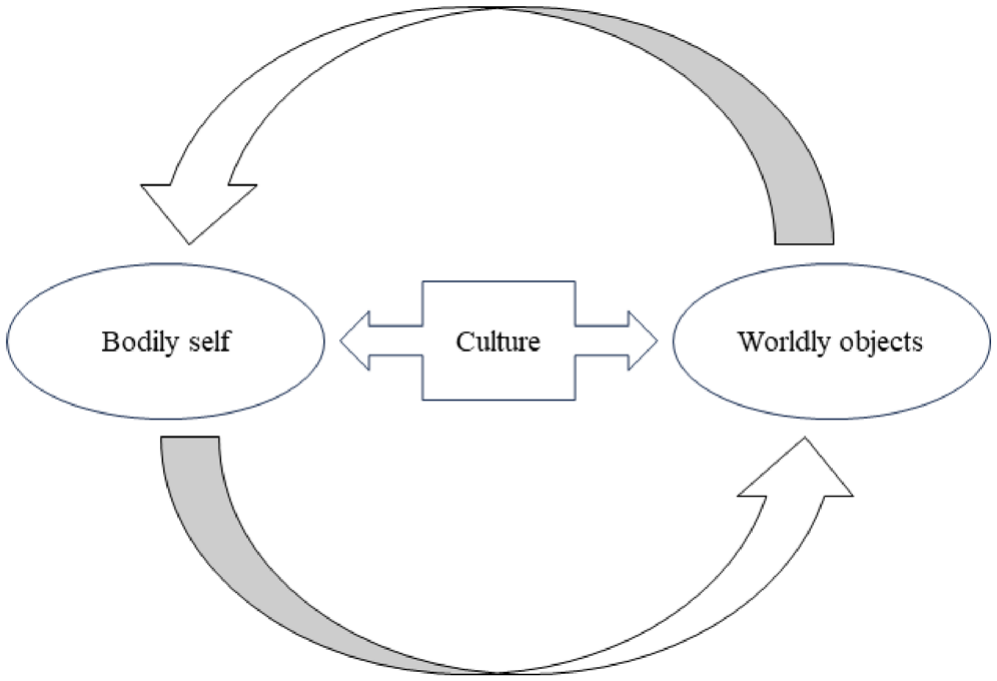
Subject–object circular relationship underlying attributional processes of meaning

Therefore, meaning attribution processing is shaped (both evolutionarily and developmentally) within a co-constitutive intertwining of brains, bodies, and things, in which “things” (in the broader sense of material forms, socio-cultural assemblages and techniques) actively and dynamically bring forth and constrain human possibilities for action and thinking (Hodder, 2011; Malafouris, 2019) (shown in Fig. 1). It is such an embodied and socio-cultural unity of the self and worldly things to color our practical immersion in the life-world with a feeling of habituality or familiarity (Fuchs & Schlimme, 2009).

The net of recursive relationships between our mind and material culture is malleable, due to the high plasticity of both brain and culture and thus context dependent, being irreducibly situated in space and time (Malafouris, 2015). This is even more true since humans are “ultra” cultural (Nielsen & Haun, 2016), as they live in and depend on culturally constructed niches accumulated and modified over generations, which actively contribute to shape the developmental plasticity of the human brain (Legare, 2017; Murren et al., 2015). In particular, the human association cortex, relatively unconstrained in its connectivity (Buckner & Krienen, 2013), late developing (Hill et al., 2010), and phylogenetically recent (Mantini et al., 2013), is highly dependent on bio-cultural feedback mechanisms (Heyes, 2003). Within a dynamically complex gene-culture coevolution (Creanza et al., 2017), these plastic developmental processes also played a pivotal role in the evolution of the human capacity for symbolic representation and language (Hutchins, 2008; Tattersall, 2016). In fact, within increasingly large niches of tool-making cultures, a widespread network of sensorimotor connections, which had already undergone long-term refinement for tool-making and gestural communication, was extensively exploited for language processing, while maintaining its original function (Glenberg & Gallese, 2012; Jirak et al., 2010; Pulvermüller, 2005; Stout & Hecht, 2017). This brought to a global reconfiguration of brain’s connectivity patterns and spontaneous rhythmicity (Murphy & Benítez-Burraco, 2017), marking the split from our ancestors around 100,000 years ago (Tattersall, 2017).

The link between language and symbolic activity is so close that we can infer the evolutionary pathways toward a complex syntactic language from the archaeological records of symbolic activity in material culture (Mellars, 2004). Actually, during evolution language served the function of “neuroenhancement” in the acquisition and representation of symbolic concepts (Borghi et al., 2022). Consistently, symbolic representations are developmentally acquired and mastered through language, which acts as a “self-constructed cognitive niche” (Clark, 2006).

Overall, through language humans acquired the unique property to dissect and recombine their animate and inanimate surroundings into a mass of intangible symbols, disengaged from the immediate contingencies of the natural environment, ending up living in self-constructed worlds of symbolic meanings (Renfrew et al., 2008a, b). Archaeological records show that from the upper Paleolithic, around 50,000 years ago, every aspect of reality (including the concept of self, others, and the world) was pervasively completed by symbolic representations (White, 1992), with a contagious spread in the material culture of the final Epipaleolithic period and earliest Neolithic, around 11,000 B.C. (Watkins, 2005). The experience of spirituality and of the sacred probably emerged simultaneously with the symbolic transfiguration of reality, when material objects started to signify something beyond themselves (concerning a more fundamental or deeper level of reality) (Donald, 2005; Rappaport, 1999). In fact, the first clear evidence of the emergence of religious beliefs parallels the flourishing of symbolically mediated material culture (Henshilwood, 2009; Tripp et al., 2014). In this connection, ritual behavior may have played a pivotal role in favoring those co-evolutionary processes among social brain size, tool-making industry, and gestural motor control, without which symbolic activity and spirituality could not have evolved (Donald, 2005). On the one hand, ritual behavior through its role in promoting motor synchronization and intra-group communication (Reddish et al., 2013; Sosis, 2000) represented the social glue for increasingly larger eco-cultural niches (Tonna et al., 2019, 2020). On the other, multiple social–emotional signals conveyed during ritual performances (involving attention, emotion, and arousal) may have turned early symbolic representations into shared religious beliefs (Deeley, 2004). In a circular way, complex symbolic systems, culturally coded in an established package of religious beliefs and practices, further reinforced prosocial connection in a long-term, cultural evolutionary process (Norenzayan et al., 2016). Therefore, a complex intertwining of brains, bodies, things, and cultural practices represented the scaffolding for the evolution of human symbolization and the sacred (Renfrew et al., 2008a, b).

Since then, our pre-reflective, bodily mode of experience (*bodily self*) became inextricably intertwined with symbolic/cultural narratives and practices (*symbolic self*) (Kirmayer & Ramstead, 2017). Humans are in fact anchored to their bodily ground of experience, while being “suspended in webs of significance” (Geertz, 1973), with the unique possibility to move freely between these two poles, i.e., from a bodily immersion into reality (embodied immediacy) to its complete disengagement (symbolic mediacy) (Henderson, 1971).

## The Neurobiology of Symbols

The fine calibration between the embodied and the symbolic interaction with the environment is made possible by their fundamental continuity (Fuchs, 2016). Concepts in fact are located on a representational continuum that ranges from more concrete (for those related to perceptible objects) to more abstract (for those with no tangible referent) (Barsalou et al., 2018). This continuity is rooted in sensorimotor grounding, which, while remaining the substrate of self-embodiment processing, was plastically re-adapted for language processing (Pulvermüller, 2005; Tonna et al., 2023a). Self-embodiment processing in fact relies on an integrated, multi-level sensorimotor network, developmentally reverberating in different domains such as somatosensory perception, fluidity and grace of movements, and sense of agency (Gallese & Ferri, 2014; Park & Baxter, 2022). The very same sensorimotor system was extensively reused for language during human speciation (Aboitiz & García, 1997; Stout & Hecht, 2017), along a gradient from concrete sensorimotor functions to abstract, domain-general processing (Stout et al., 2008).

In this connection, different functions (from sensorimotor processing to concrete representations up to more abstract concepts) emerge from the flexible combining of the same neural structures in different coalitions (Mazzuca et al., 2021). This is made possible by specific properties of sensorimotor system: (1) context dependence (specific functional configurations are the result of specific bio-cultural inputs); (2) intrinsic neural dynamicity (novel functions emerge from the mutual plastic modulation between the nodes of the same network) (Anderson, 2010). In this respect, during ontogeny, many complex developmental (psychosocial, bio-cultural) mechanisms, acting on fine-tuning processes of neural circuitry, contribute calibrating the neural pathways and functional outcome of this integrated sensorimotor-language system. There is evidence for example that socio-cultural practices influence affordance activation (i.e., the motor recruitment during the observation of graspable objects), thus shaping the embodied meaning we bestow to objects (Rietveld, 2014). Of utmost importance, the flexible and context-dependent character of sensorimotor grounding is pushed up to convey abstract concepts and symbolic representations (Borghi et al., 2022). In this regard, during development, socio-cultural pressures drive the formation of relatively unconstrained neural connectivity, with the emergence of “noncanonical” association networks, which are primarily connected with each other than with more constrained sensorimotor inputs (Buckner & Krienen, 2013). For example, along a gradient of semantic abstraction of language, sensorimotor circuits open up capturing higher-order conjunctions, which are then neurally stored in specific convergent zones (Meteyard et al., 2012). In other words, at increasing levels of symbolization, concepts are gradually freed from rigid sensorimotor constraints in favor of looser connections, whose degree of neural “relaxation” is shaped by socio-cultural context (Mazzuca et al., 2021).

Evolutionary heterochronic mechanisms leading to delay in timing and rates of development (also referred to as “neoteny”) may have played a pivotal role in maintaining the high levels of sensorimotor plasticity necessary to be re-adapted for novel, higher-order functions, such as symbolic activity (Brüne, 2000; Bufill et al., 2011). As a downside, recently evolved and less constrained neural configurations are less resilient to various developmental damages, due to a lack of compensatory mechanisms (Toro et al., 2010). In this respect, culturally determined developmental processes may have a coping effect or, on the contrary, trigger an underlying vulnerability (Kirmayer & Ryder, 2016).

**Fig. 2:**
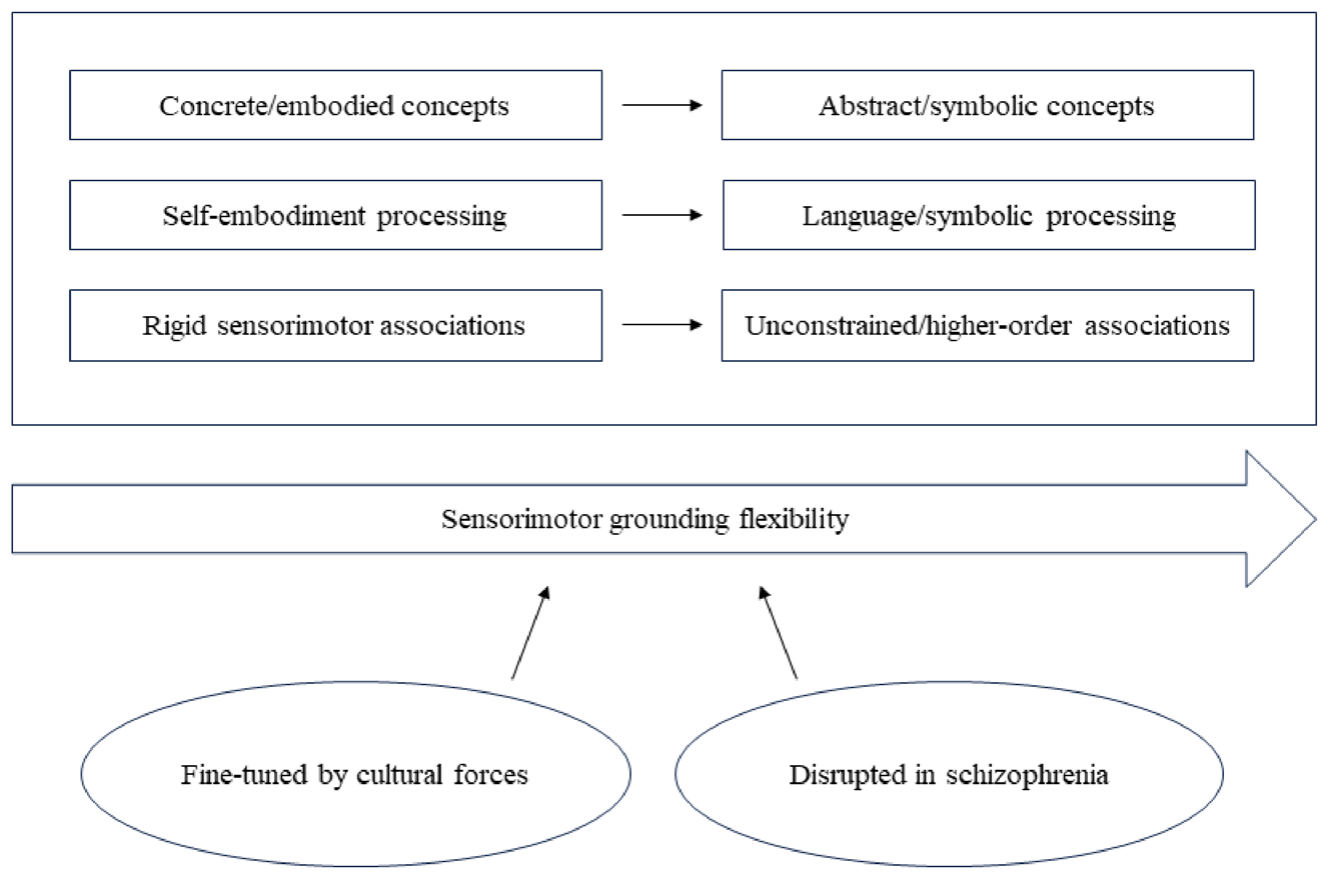
Sensorimotor grounding from embodiment to symbolic processing

Therefore, along a continuity from embodied to symbolic mode of experience, sensorimotor connectivity is reshaped from phylogenetically constructed patterns to developmentally driven organizations (Heyes, 2003). On the one hand, culture represents the background in which embodied components are merged with more abstract concepts into a unique, coherent perceptual field; on the other, it is the developmental neural dynamicity of sensorimotor system to allow plastic alignment to recurring socio-cultural environment (Murren et al., 2015). However, because of its “extended, distributed, embodied and culturally mediated character” (Renfrew et al., 2008a, b), the human mind is inherently vulnerable. In fact, the more it relies on unconstrained connections, the more it is exposed to a complete disconnection from its sensorimotor grounding. In this regard, schizophrenia might represent the catastrophic failure of such fine-grained balancing mechanisms at the basis of our symbolic, yet embodied, activity (shown in Fig. 2).

## Sensorimotor Disconnection in Schizophrenia

Schizophrenia is a severe neurodevelopmental disorder (Rapoport et al., 2012), specifically linked to the evolution of language and higher symbolic activities (DeLisi, 2021) [the “price” humans paid for language, according to Timothy Crow (2000)] and underpinned by a diffuse neural dysconnectivity (Friston et al., 2016). In this respect, great emphasis has been placed in recent years on a specific disruption of multisensory and sensorimotor grounding (Park & Baxter, 2022).

The integration of different sensory modalities into a coherent perceptual whole is tuned to species-specific spatiotemporal ranges (Stevenson et al., 2014), orchestrated by the dynamics of brain’s spontaneous activity (Northoff & Stanghellini, 2016). Therefore, these particular integration patterns impose evolutionary conserved sensorimotor constraints that ground our engagement in the world (Borghi et al., 2022), yet maintaining plastic properties, especially in developmental years (de Klerk et al., 2021; Powers et al., 2009).

In the schizophrenia spectrum, there is evidence for a widened temporal window of integration (i.e., a reduced ability to segregate stimuli in time) (Di Cosmo et al., 2021), as well as for a reduced and less demarcated peripersonal space (PPS) (i.e., a more restricted and blurred space surrounding our body within which sensory stimuli are normally integrated) (Ferroni et al., 2020, 2022). As a result, patients with schizophrenia show an overall impaired ability to integrate and process sensory stimuli in order to execute a contextually appropriate motor action (Carment et al., 2019). An early and diffuse disruption in sensorimotor development and maturation in at-risk individuals manifests as reliable motor biomarkers from the first 2 years of life, such as gait and balance deficits, delayed gross motor milestones and dyscoordination (Walther & Strik, 2012). Remarkably, in the schizophrenia spectrum, sensorimotor impairment appears to specifically aggregate with both language disturbances (Schiffman et al., 2015) and subjective bodily experiences (Tonna et al., 2023b) into unique self-motor-language phenotypes, thus suggesting a disruption in the intertwined developmental pathways linking motor, self-embodiment, and higher cognitive processing (Poletti & Raballo, 2022). In this connection, we have hypothesized elsewhere that in schizophrenia the diffuse perturbation of sensorimotor grounding may be brought back to a developmental connectivity rearrangement excessively unbalanced toward language and symbolic processing, and therefore overly untethered from its sensorimotor constraints, leading to a hyper-symbolic and disembodied self (for a summary see: Tonna et al., 2023a). In this vein, the “symbolic explosion” (Mithen, 1996), which saw the birth of human uniqueness, had its counterpart in exposing few unfortunate individuals to a catastrophic mismatch between embodied and symbolic modes of experience.

## Schizophrenia Psychopathology and Ethnoarchaeology: A Comparative Approach

If this is the case, we should find invariant subjective structures, based on the uniquely human property of symbolic representation, behind specific psychopathological experiences in individuals with schizophrenia and in different cultural manifestations from archaeological and anthropological records, pathologically over-expressed in the former, while finely attuned with socio-cultural context in the latter. Indeed, the few comparative studies available (Dein & Littlewood, 2011; Parnas & Henriksen, 2014; Storch, 1924) have repeatedly highlighted the striking similarity in both formal features and contents of symbolic activity as expressed in schizophrenia patients and human cultures.

The disruption of self-embodiment processing (self-disembodiment) is subjectively felt in high-risk individuals long before the clinical onset as a diminished sense of existing as embodied subjects, vitally immersed in the world and authors of their own actions. On the other hand, these patients tend to be absorbed in empty metaphysic or pseudo-philosophical concerns, which outweigh everyday life occupations (Parnas & Henriksen, 2014). In a similar vein, schizophrenia delusions typically respond to “ontological” urges, as they dramatically question the fundamental nature of reality and our ultimate predicament of existence (Sass, 1992a, b). Therefore, the detachment from the embodied ground of experience seems to allow a flourishing symbolic activity to emerge, in the attempt, ubiquitous in human cultures, to make sense to a world beyond its immediate physical characteristics (Watkins, 2005).

However, it is in the precursor stages of schizophrenia delusion (often referred to as “*delusional atmosphere*”) (Conrad, 1958; Jaspers, 2013) that the human predisposition to symbolization is dramatically emphasized. During these transient, clinically elusive (not yet psychotic) phases, within a radical alienation of the environment, objects may lose their familiar, pragmatic meaning to acquire an indefinite, uncanny quality. The erosion of the common-sense understanding of things is experienced as a kind of incoercible perplexity (Humpston, 2014) or unfamiliarity (in German: *Unheimlichkeit*) (Škodlar & Henriksen, 2019), by which patients sought to articulate their pervasive feeling of “not being at home” (unheimlich literally means “nonhomely”). This actually implies a radical change in perception itself, as certain aspects or peculiarities of perceived objects (also referred to as “physiognomic” or “essential” properties) stand out from the perceptual field in such a way that they achieve a special salience (Conrad, 1958; Matussek, 1952). These particular features become “symbolic,” insofar as they start evoking a multiplicity of new significances, undermining the perceptual coherence normally built upon our practical possibilities for action (Chemero, 2019).

In other words, the suspension of common, everyday meanings, which is normally rooted in our embodied enactment, allows novel, idiosyncratic affordances to gain salience (Kapur, 2003) so that objects may become symbolically pregnant, opening up a plurality of meanings, in a sort of polysemic diffusion (Peirce, 1991). As psychosis advances, ordinary worldly relations can collapse into a confused abundance of newly emerging symbolic connections (Storch, 1924).

These psychopathological experiences raise in an acute form the “constructed” character of symbolic attribution in the human species, which fits well with ethnoarchaeological accounts of human symbolic material. Within this framework in fact, symbolic properties of material culture are viewed as perceptive fragments with emergent qualities (such as peculiar color, shape, size), which are inherently arbitrary, but temporarily assembled and felt as particularly meaningful in the immediate moment of experience (Robb, 1998).

This sort of hyper-symbolic diffusion in pre-delusional stages ceases when things start regaining a novel, unambiguous meaning, albeit delusional. The paradigmatic example is represented by “*delusional perception,*” a nearly pathognomonic [“primary” (Jaspers, 2013); “first-rank” (Schneider, 2007)] symptom of schizophrenia. In this phenomenon, a common object, which is real (in that the patient recognizes and will acknowledge the meaning that it has for others), nonetheless acquires a new, mysterious, and bewildering revelatory significance from “a higher reality.” The underlying experiential structure is a sudden, idiosyncratic, and self-referential meaning, pertaining a deeper level of reality, embedded in specific perceptual peculiarities of the object (Matussek, 1952) and passively superimposed to the subject in the form of revelation (*apophany*) (Conrad, 1958; Schneider, 2007). A patient with schizophrenia may state for example that a chair in a room, while remaining a chair, nevertheless it signifies the descent of the Messiah to the earth (Arieti, 1970).

Strikingly similar formal features are described in “hierophany” (manifestation of the sacred) (Eliade, 1959): in this ubiquitous cultural experience, ordinary objects can become symbolically pregnant, evoking a novel (“sacred”), extra-ordinary (“wholly other”) significance to the subject in the form of revelation. A stone or a tree for example, by virtue of certain features (in form, color, location, and so forth), while maintaining their practical meaning, nonetheless reveal or stands for something else, totally different, more real, sacred in fact (Eliade, 1959).

The formal structure in both phenomena presents the following features:

1. The novel meaning, inherent in specific perceptive peculiarities of the object, emerges as immediate and absolute certainty (*epiphany*), even though not replacing the ordinary one (Nielsen et al., 2022). The maintenance of the ordinary meaning alongside the delusional one (experienced as more real or fundamental) may favor a specific condition (referred to as “double bookkeeping” Bleuler, 1911), in which delusional world and socially shared world can exist peacefully side-by-side. In this case, the two attitudes are kept separated and patients do not call into question the everyday social world while being absorbed in their psychotic experiences (Parnas et al., 2021).
2. The emergence of symbolic properties from perceptual features is subjectively perceived in a dynamic and passive way; i.e., it is forced upon the subject. In the prodromal phases of psychosis for example, objects may unveil new, enigmatic, bewildering forces, subtly perceived by the patient in a passive and self-referential way (Conrad, 1958); single aspects or details are charged with hidden, prominent energies, which can catch and even penetrate the subject, parallel to the dissolution of stable and definitive self boundaries (Storch, 1924). In this vein, things can be experienced as “nodes” of supernatural flows of energy, which connect objects to each other and fall on the patient by merging with him (Fuchs, 2005). These subjective experiences may then crystallize into the characteristic delusions of influence [Transitivism (Bleuler, 1911); First Rank Symptoms (Schneider, 2007)], in which patients’ thoughts, feelings, impulses or behaviors are concretely and physically influenced or manipulated by a mass of forces, powers and fluxes (Eckstein et al., 2022).

Not dissimilarly, cultural symbols are dynamically perceived as flows of energy and as such they channel and instantiate overwhelming forces (Hodder, 2014). Some objects for example, by virtue of certain properties, especially if new or unusual, can be imbued with sacred forces (*mana* in ethnological literature) and “*numinous*” energies, in all their ambivalence both attractive and repelling, whose brute power must be carefully managed or kept at distance so as not to be oppressed (Otto, 1923). According to Lévi-Strauss (1987), it is symbolic proliferation itself to be subjectively felt and culturally explained as forces concentrated or encapsulated in things or pulses of energy emanated from them. In human cultures, supernatural powers and forces may also be incorporated in specific manufactured items (figurines, plastered skulls, and so forth) so to harness and control their fluxes. Their frequently magnified physiognomic features (see for example the exaggerated anatomic characteristics of the “Venus figurines”) are plenty of these vital flows, which have to be captured and recirculated in order to renovate and strengthen reality (Hodder & Hutson, 2003).

It should be noted that any aspect of reality may potentially become symbolic, even included human physiology and body: the breath, blood, pulse, semen, and body warmth of ordinary human beings can all become signs of the presence of supernatural forces (Csordas, 1994). In schizophrenia, this process of symbolization of bodily parts may be pushed to the extremes: for example, Storch (1924) described a patient whose navel had become a doorway to other places; a patient of mine had to protect his sacred semen because the forces contained in it could originate entire cosmos. Actually, in patients with schizophrenia symbolic activity seems to function in a vacuum, where symbols may evoke other symbols in an endless chain, just driven by imaginative juxtaposition (Storch, 1924): a patient of mine for example was used to place two seeds on her windowsill as symbols of her rebirth. After seeing two birds come to pick them up, the birds first became her parents (because they gave birth to her), then also the Holy Spirit (who gave rebirth to humanity).

To sum up, a cross-disciplinary comparison between psychopathology to ethnoarchaeology (Table 1) highlights, we suggest, essential structural features of human consciousness, i.e., the uniquely human property of symbolic thought. Symbolic meaning stems from the emergence of salient perceptive fragments, inherent to the object but standing out from the perceptual field and it is experienced: (1) as immediate certainty, though not replacing the common-sense meaning; (2) in a dynamic and passive way.

**Table 1:** Structural comparison of symbolic activity in schizophrenia psychopathology and in cultural manifestations.

Schizophrenia symbolization	Cultural symbolization
Overly untethered sensorimotor connections impairing self-embodiment processing	Degree of sensorimotor flexibility tuned to cultural background
Suspension of common-sense meaning and openness to a diffusion of significance	Practical, common meaning is parenthesized
“Physiognomic” or “essential” properties	Perceptive fragments with “emergent” qualities
Novel meaning, embedded in the object and forced upon the subject in the form of revelation (*apophany*)	Novel (“sacred”), extra-ordinary (“wholly other”) significance superimposed to the subject (*epiphany*)
Passive and self-referential way: prominent energies catch the subject (up to passivity delusions)	Symbolic proliferation passively felt as flows of energy emanating from the object
Dissolution of the self with blurred boundaries	Maintenance of a stable and coherent self
Potentially endless chain of recalled symbolic meanings	Symbolic signification is constrained by socio-cultural framework
The meaning stems from a fracture between the embodied and the socio-cultural frame of reference	The meaning falls within an integrated embodied and socio-cultural frame of reference

Human symbolism is intimately connected with the experience of spirituality, since through symbolic transformation an ordinary object may stand for other than what it appears in a revelatory manner, thus as such inherently “transcendent.” The crucial difference is that in cultural manifestations, symbols derive their meaning from their relations to other symbols within a specific cultural system. This web of symbols is bodily grounded (Mazzuca, et al., 2021) and intersubjectively fine-tuned (Deacon, 1997; Hodder & Hutson, 2003) and represents the obvious, taken-for-granted frame of reference within which our worldly experience is ordered. Instead, in schizophrenia, symbolic activity is affected by a weakening of embodiment processing (the loss of “the natural perceptual context” according to Matussek, 1987), due to a developmental disruption of sensorimotor grounding. In that case, symbolic meaning attribution loses its pre-reflective, tacit character to emerge, dissected, or amplified, in all its problematic nature. For this very reason, however, these basic phenomena elicit an active effort of the individual with schizophrenia to compensate, make sense, or adapt to his/her uncanny, almost ineffable experiences (Stanghellini et al., 2022). The result is a mixing of “act and affliction,” i.e., a complex interplay between passively determined basic disturbances and preserved forms of agency on the patient’s part (Sass, 1992a, b) or, as Wyrsch puts it (Wyrsch, 1949), the active way the person takes position in front of them. This active role is shaped by the individual’s biography, dispositions, and character peculiarities and unfolds within a given historical and socio-cultural context (Stanghellini et al., 2022). Consequently, patients’ process of elaboration of their basic experiences is imbued with cultural values, beliefs, and practices, which actively guide adaptive responses and coping mechanisms and shape the contents of delusional explanations [the “psychotic work” according to Henry Ey (1973)].

As a main limitation, this contribution is not a systematic review per se, but takes a comprehensive approach to surveying the literature that the authors considered as conducive to address the main hypothesis proposed. Additionally, in order to investigate human symbolic activity, our account of psychopathological symptoms was mainly focused on “reality distortion” dimension. This has left out important schizophrenia symptom dimensions, such as negative symptoms, which actually represent a core feature of the disorder (Loch, 2019; Marchesi et al., 2014).

## Conclusion

In the recent years, the question of the evolutionary emergence of human mindedness has been debated from different perspectives (Noble & Davidson, 1996). However, to our knowledge, a psychopathological approach to the issue is lacking. This is unfortunate since psychiatric conditions allow the emergence, in a distorted or magnified way, of invariant modes of experiences, deeply rooted in our phylogeny and developmentally shaped by bio-cultural feedbacks (Kirmayer & Ryder, 2016). In this vein, a fine-grained psychopathology, aimed at capturing qualitative modifications of subjective experiences, may help illuminate such basic structures of human subjectivity, from which interdisciplinary research could be guided (Northoff & Stanghellini, 2016).

In this connection, symbolic thought, along with a fully developed language, arises evolutionarily and developmentally within a circular relationship between enacting bodies and worldly objects, grounded in sensorimotor connectivity. Within this network, the fine-tuned calibration between constrained sensorimotor connections (responsible for self-embodiment processing) and untethered, higher-order conjunctions (responsible for more abstract and symbolic processing) is epigenetically malleable, context dependent, and plastically neuromodulated. The pattern of neural flexibility varies across cultures and epochs, so to be attuned with its specific eco-cultural context. Instead, in schizophrenia, sensorimotor grounding is pushed toward an excessively unconstrained connectivity, falling off catastrophically. Therefore, the profound distortion of self/world relation in individuals with schizophrenia may be evolutionarily viewed as a failed trade-off between symbolic and embodiment processing. In this perspective, however, schizophrenia psychopathology, lying at the interface of brain–culture interaction, may contribute to elucidate, within an interdisciplinary framework, the basic experiential structures underlying symbolic representation and thus to unravel the bio-cultural pathways to human symbolic evolution.

## References

[CR1] Aboitiz, F., & García, V. R. (1997). The evolutionary origin of the language areas in the human brain. A neuroanatomical perspective. *Brain Research. Brain Research Reviews,**25*(3), 381–396. 10.1016/s0165-0173(97)00053-2 9495565 10.1016/s0165-0173(97)00053-2

[CR2] Anderson, M. L. (2010). Neural reuse: A fundamental organizational principle of the brain. *The Behavioral and Brain Sciences,**33*(4), 245–266. 10.1017/S0140525X10000853 20964882 10.1017/S0140525X10000853

[CR3] Arieti, S. (1970). The concept of Schizophrenia. In R. Cancro (Ed.), *The schizophrenic reactions. *Brunner Mazel Inc.

[CR4] Barsalou, L. W., Dutriaux, L., & Scheepers, C. (2018). Moving beyond the distinction between concrete and abstract concepts. *Philosophical Transactions of the Royal Society of London. Series b, Biological Sciences*. 10.1098/rstb.2017.0144 10.1098/rstb.2017.0144 PMC6015837 29915012

[CR5] Bentley, R. A., & O’Brien, M. J. (2012). Cultural evolutionary tipping points in the storage and transmission of information. *Frontiers in Psychology,**3*, 569. 10.3389/fpsyg.2012.00569 23267338 10.3389/fpsyg.2012.00569 PMC3525879

[CR6] Bleuler, E. (1911). *Dementia praecox, oder Gruppe der Schizophrenien*. Deuticke. 10.1192/bjp.149.5.661 3545358

[CR7] Borghi, A. M., Shaki, S., & Fischer, M. H. (2022). Abstract concepts: External influences, internal constraints, and methodological issues. *Psychological Research Psychologische Forschung,**86*(8), 2370–2388. 10.1007/s00426-022-01698-4 35788903 10.1007/s00426-022-01698-4 PMC9674746

[CR8] Brüne, M. (2000). Neoteny, psychiatric disorders and the social brain: hypotheses on heterochrony and the modularity of the mind. *Anthropology & Medicine,**7*, 301–318. 10.1080/713650607.

[CR9] Buckner, R. L., & Krienen, F. M. (2013). The evolution of distributed association networks in the human brain. *Trends in Cognitive Sciences,**17*(12), 648–665. 10.1016/j.tics.2013.09.017 24210963 10.1016/j.tics.2013.09.017

[CR10] Bufill, E., Agustí, J., & Blesa, R. (2011). Human neoteny revisited: The case of synaptic plasticity. *American Journal of Human Biology,**23*(6), 729–739. 10.1002/ajhb.21225 21957070 10.1002/ajhb.21225

[CR11] Carment, L., Dupin, L., Guedj, L., Térémetz, M., Krebs, M.-O., Cuenca, M., Maier, M. A., Amado, I., & Lindberg, P. G. (2019). Impaired attentional modulation of sensorimotor control and cortical excitability in schizophrenia. *Brain: A Journal of Neurology,**142*(7), 2149–2164. 10.1093/brain/awz127 31099820 10.1093/brain/awz127 PMC6598624

[CR12] Chemero, A. (2019). *Radical embodied cognitive science*. MIT Press.

[CR13] Clark, A. (2006). Language, embodiment, and the cognitive niche. *Trends in Cognitive Sciences,**10*(8), 370–374. 10.1016/j.tics.2006.06.012 16843701 10.1016/j.tics.2006.06.012

[CR14] Conrad, K. (1958). *Die beginnende schizophrenie*. Thieme.

[CR15] Creanza, N., Kolodny, O., & Feldman, M. W. (2017). Cultural evolutionary theory: How culture evolves and why it matters. *Proceedings of the National Academy of Sciences of the United States of America,**114*(30), 7782–7789. 10.1073/pnas.1620732114 28739941 10.1073/pnas.1620732114 PMC5544263

[CR16] Crow, T. J. (2000). Schizophrenia as the price that homo sapiens pays for language: A resolution of the central paradox in the origin of the species. *Brain Research. Brain Research Reviews,**31*(2–3), 118–129. 10.1016/s0165-0173(99)00029-6 10719140 10.1016/s0165-0173(99)00029-6

[CR17] Csordas, T. J. (1994). *Embodiment and experience: The existential ground of culture and self*. Cambridge University Press.

[CR18] de Klerk, C. C. J. M., Filippetti, M. L., & Rigato, S. (2021). The development of body representations: An associative learning account. *Proceedings. Biological Sciences*. 10.1098/rspb.2021.0070 10.1098/rspb.2021.0070 PMC8079995 33906399

[CR19] de Vries, R., Heering, H. D., Postmes, L., Goedhart, S., Sno, H. N., & de Haan, L. (2013). Self-disturbance in schizophrenia: A phenomenological approach to better understand our patients. *The Primary Care Companion for CNS Disorders*. 10.4088/PCC.12m01382 10.4088/PCC.12m01382 PMC3661330 23724352

[CR20] Deacon, T. W. (1997). *The symbolic species: The co-evolution of language and the brain*. W. W. Norton & Company.

[CR21] Dein, S., & Littlewood, R. (2011). Religion and psychosis: A common evolutionary trajectory? *Transcultural Psychiatry,**48*(3), 318–335. 10.1177/1363461511402723 21742955 10.1177/1363461511402723

[CR22] Deeley, P. (2004). The religious brain. *Anthropology & Medicine,**11*(3), 245–267. 10.1080/1364847042000296554 26868319 10.1080/1364847042000296554

[CR23] DeLisi, L. E. (2021). Historical pursuits of the language pathway hypothesis of schizophrenia. *NPJ Schizophrenia,**7*(1), 53. 10.1038/s41537-021-00182-z 34753947 10.1038/s41537-021-00182-z PMC8578658

[CR24] Di Cosmo, G., Costantini, M., Ambrosini, E., Salone, A., Martinotti, G., Corbo, M., Di Giannantonio, M., & Ferri, F. (2021). Body-environment integration: Temporal processing of tactile and auditory inputs along the schizophrenia continuum. *Journal of Psychiatric Research,**134*, 208–214. 10.1016/j.jpsychires.2020.12.034 33418447 10.1016/j.jpsychires.2020.12.034

[CR25] Donald, M. (2005). Imitation and mimesis. In S. Hurley & N. Chater (Eds.), *Perspectives on imitation: From neuroscience to social science. Imitation, human development, and culture* (Vol. 2, pp. 283–300). MIT Press.

[CR26] Eckstein, K. N., Rosenbaum, D., Zehender, N., Pleiss, S., Platzbecker, S., Martinelli, A., Herrmann, M. L., & Wildgruber, D. (2022). Induced feelings of external influence during instructed imaginations in healthy subjects. *Frontiers in Psychology*. 10.3389/fpsyg.2022.1005479 10.3389/fpsyg.2022.1005479 PMC9664387 36389532

[CR27] Eliade, M. (1959). *The sacred and the profane: The nature of religion*. Harcourt, Brace & World.

[CR28] Ferroni, F., Ardizzi, M., Ferri, F., Tesanovic, A., Langiulli, N., Tonna, M., Marchesi, C., & Gallese, V. (2020). Schizotypy and individual differences in peripersonal space plasticity. *Neuropsychologia*. 10.1016/j.neuropsychologia.2020.107579 10.1016/j.neuropsychologia.2020.107579 32758552

[CR29] Ey, H. (1973). *Traité des hallucinations Tome I et II*. Masson.

[CR30] Ferroni, F., Ardizzi, M., Magnani, F., Ferri, F., Langiulli, N., Rastelli, F., Lucarini, V., Giustozzi, F., Volpe, R., Marchesi, C., Tonna, M., & Gallese, V. (2022). Tool-use extends peripersonal space boundaries in schizophrenic patients. *Schizophrenia Bulletin,**48*(5), 1085–1093. 10.1093/schbul/sbac067 35708490 10.1093/schbul/sbac067 PMC9434469

[CR31] Friston, K., Brown, H. R., Siemerkus, J., & Stephan, K. E. (2016). The dysconnection hypothesis. *Schizophrenia Research,**176*(2–3), 83–94. 10.1016/j.schres.2016.07.014 27450778 10.1016/j.schres.2016.07.014 PMC5147460

[CR32] Fuchs, T. (2005). Delusional mood and delusional perception-A phenomenological analysis. *Psychopathology,**38*(3), 133–139. 10.1159/000085843 15905636 10.1159/000085843

[CR33] Fuchs, T. (2016). The embodied development of language. In G. Etzelmüller & C. Tewes (Eds.), *Embodiment in evolution and culture* (pp. 107–128). Mohr Siebeck Verlag.

[CR34] Fuchs, T., & Schlimme, J. E. (2009). Embodiment and psychopathology: A phenomenological perspective. *Current Opinion in Psychiatry,**22*(6), 570–575. 10.1097/YCO.0b013e3283318e5c 19730373 10.1097/YCO.0b013e3283318e5c

[CR35] Gallagher, S. (2017). *Enactivist interventions: Rethinking the mind*. Oxford University Press.

[CR36] Gallese, V., & Ferri, F. (2014). Psychopathology of the bodily self and the brain: The case of schizophrenia. *Psychopathology,**47*(6), 357–364. 10.1159/000365638 25359279 10.1159/000365638

[CR37] Geertz, C. (1973). *The interpretation of cultures: Selected essays*. Basic Books Inc.

[CR38] Glenberg, A. M., & Gallese, V. (2012). Action-based language: A theory of language acquisition, comprehension, and production. *Cortex: A Journal Devoted to the Study of the Nervous System and Behavior,**48*(7), 905–922. 10.1016/j.cortex.2011.04.010 21601842 10.1016/j.cortex.2011.04.010

[CR39] Henderson, E. H. (1971). Homo Symbolicus. *Man and World,**4*(2), 131–150. 10.1007/BF01248599

[CR40] Henshilwood, C. S. (2009). *Becoming human: Innovation in prehistoric material and spiritual cultures*. Cambridge University Press.

[CR41] Heyes, C. (2003). Four routes of cognitive evolution. *Psychological Review,**110*(4), 713–727. 10.1037/0033-295X.110.4.713 14599239 10.1037/0033-295X.110.4.713

[CR42] Hill, J., Inder, T., Neil, J., Dierker, D., Harwell, J., & Van Essen, D. (2010). Similar patterns of cortical expansion during human development and evolution. *Proceedings of the National Academy of Sciences of the United States of America,**107*(29), 13135–13140. 10.1073/pnas.1001229107 20624964 10.1073/pnas.1001229107 PMC2919958

[CR43] Hodder, I. (2011). Human-thing entanglement: Towards an integrated archaeological perspective. *Journal of the Royal Anthropological Institute,**17*(1), 154–177. 10.1111/j.1467-9655.2010.01674.x.

[CR44] Hodder, I. (2014). *Symbols in action: Ethnoarchaeological studies of material culture*. Cambridge University Press.

[CR45] Hodder, I., & Hutson, S. (2003). *Reading the past: Current approaches to interpretation in archaeology* (3rd ed.). Cambridge University Press. 10.1017/CBO9780511814211

[CR46] Humpston, C. S. (2014). Perplexity and meaning: Toward a phenomenological «core» of psychotic experiences. *Schizophrenia Bulletin,**40*(2), 240–243. 10.1093/schbul/sbt074 23698126 10.1093/schbul/sbt074 PMC3932087

[CR47] Hutchins, E. (2008). The role of cultural practices in the emergence of modern human intelligence. *Philosophical Transactions of the Royal Society of London. Series b, Biological Sciences,**363*(1499), 2011–2019. 10.1098/rstb.2008.0003 18292065 10.1098/rstb.2008.0003 PMC2606696

[CR48] Insel, T. R. (2010). Rethinking schizophrenia. *Nature,**468*(7321), 187–193. 10.1038/nature09552 21068826 10.1038/nature09552

[CR49] Jaspers, K. (2013). *Allgemeine psychopathologie*. Springer.

[CR50] Jirak, D., Menz, M. M., Buccino, G., Borghi, A. M., & Binkofski, F. (2010). Grasping language-A short story on embodiment. *Consciousness and Cognition,**19*(3), 711–720. 10.1016/j.concog.2010.06.020 20739194 10.1016/j.concog.2010.06.020

[CR51] Kapur, S. (2003). Psychosis as a state of aberrant salience: A framework linking biology, phenomenology, and pharmacology in schizophrenia. *The American Journal of Psychiatry,**160*(1), 13–23. 10.1176/appi.ajp.160.1.13 12505794 10.1176/appi.ajp.160.1.13

[CR52] Kirmayer, L. J., & Ramstead, M. J. D. (2017). Embodiment and enactment in cultural psychiatry. In C. Durt, T. Fuchs, & C. Tewes (Eds.), *Embodiment, enaction, and culture: Investigating the constitution of the shared world* (pp. 397–422). Boston Review.

[CR53] Kirmayer, L. J., & Ryder, A. G. (2016). Culture and psychopathology. *Current Opinion in Psychology,**8*, 143–148. 10.1016/j.copsyc.2015.10.020 29506790 10.1016/j.copsyc.2015.10.020

[CR54] Laland, K. N. (2017). *Darwin’s unfinished symphony: How culture made the human mind*. Princeton University Press.

[CR55] Legare, C. H. (2017). Cumulative cultural learning: Development and diversity. *Proceedings of the National Academy of Sciences of the United States of America,**114*(30), 7877–7883. 10.1073/pnas.1620743114 28739945 10.1073/pnas.1620743114 PMC5544271

[CR56] Lévi-Strauss, C. (1987). *Antropología estructural: Mito, sociedad, humanidades*. Siglo XXI.

[CR57] Loch, A. A. (2019). Schizophrenia, not a psychotic disorder: Bleuler revisited. *Frontiers of Psychiatry*. 10.3389/fpsyt.2019.00328 10.3389/fpsyt.2019.00328 PMC6526283 31133901

[CR58] Malafouris, L. (2015). Metaplasticity and the primacy of material engagement. *Time and Mind,**8*(4), 351–371. 10.1080/1751696X.2015.1111564

[CR59] Malafouris, L. (2019). Mind and material engagement. *Phenomenology and the Cognitive Sciences,**18*(1), 1–17. 10.1007/s11097-018-9606-7 31523220 10.1007/s11097-018-9606-7 PMC6713400

[CR60] Mantini, D., Corbetta, M., Romani, G. L., Orban, G. A., & Vanduffel, W. (2013). Evolutionarily nonvel functional networks in the human brain? *The Journal of Neuroscience,**33*(8), 3259–3275. 10.1523/JNEUROSCI.4392-12.2013 23426655 10.1523/JNEUROSCI.4392-12.2013 PMC6619535

[CR61] Marchesi, C., Affaticati, A., Monici, A., De Panfilis, C., Ossola, P., & Tonna, M. (2014). Predictors of symptomatic remission in patients with first-episode schizophrenia: A 16 years follow-up study. *Comprehensive Psychiatry,**55*, 778–784. 10.1016/j.comppsych.2013.12.011 24461689 10.1016/j.comppsych.2013.12.011

[CR62] Matussek, P. (1987). Studies in delusional perception. In J. Cutting & M. Sheperd (Eds.), *The clinical roots of the schizophrenia concept: Translations of seminal European contributions on schizophrenia* (pp. 89–103). Cambridge University Press.

[CR63] Matussek, P. (1952). Untersuchungen über die Wahn wahrnehmung. I. Mitteilung. Veränderungen der Wahrnehmungswelt bei beginnendem, primären Wahn. *Archiv Für Psychiatrie und Nervenkrankheiten,**189*(4), 279–319. 10.1007/BF00351194 13041256

[CR64] Mazzuca, C., Fini, C., Michalland, A. H., Falcinelli, I., Da Rold, F., Tummolini, L., & Borghi, A. M. (2021). From affordances to abstract words: The flexibility of sensorimotor grounding. *Brain Sciences,**11*(10), 1304. 10.3390/brainsci11101304 34679369 10.3390/brainsci11101304 PMC8534254

[CR65] Mellars, P. (2004). Neanderthals and the modern human colonization of Europe. *Nature,**432*(7016), 461–465. 10.1038/nature03103 15565144 10.1038/nature03103

[CR66] Meteyard, L., Cuadrado, S. R., Bahrami, B., & Vigliocco, G. (2012). Coming of age: A review of embodiment and the neuroscience of semantics. *Cortex: A Journal Devoted to the Study of the Nervous System and Behavior,**48*(7), 788–804. 10.1016/j.cortex.2010.11.002 21163473 10.1016/j.cortex.2010.11.002

[CR67] Mithen, S. (1996). *The prehistory of the mind: The cognitive origins of art*. Religion and Science. Thames and Hudson Ltd.

[CR68] Murphy, E., & Benítez-Burraco, A. (2017). Language deficits in schizophrenia and autism as related oscillatory connectomopathies: An evolutionary account. *Neuroscience and Biobehavioral Reviews,**83*, 742–764. 10.1016/j.neubiorev.2016.07.029 27475632 10.1016/j.neubiorev.2016.07.029

[CR69] Murren, C. J., Auld, J. R., Callahan, H., Ghalambor, C. K., Handelsman, C. A., Heskel, M. A., Kingsolver, J. G., Maclean, H. J., Masel, J., Maughan, H., Pfennig, D. W., Relyea, R. A., Seiter, S., Snell-Rood, E., Steiner, U. K., & Schlichting, C. D. (2015). Constraints on the evolution of phenotypic plasticity: Limits and costs of phenotype and plasticity. *Heredity,**115*(4), 293–301. 10.1038/hdy.2015.8 25690179 10.1038/hdy.2015.8 PMC4815460

[CR70] Nicholas, D., & Kramer, C. (2001). *Ethnoarchaeology in action*. Cambridge University Press.

[CR71] Nielsen, K. M., Nordgaard, J., & Henriksen, M. G. (2022). Delusional perception revisited. *Psychopathology,**55*(6), 325–334. 10.1159/000524642 35588694 10.1159/000524642

[CR72] Nielsen, M., & Haun, D. (2016). Why developmental psychology is incomplete without comparative and cross-cultural perspectives. *Philosophical Transactions of the Royal Society b: Biological Sciences*. 10.1098/rstb.2015.0071 10.1098/rstb.2015.0071 PMC4685517 26644590

[CR73] Noble, W., & Davidson, I. (1996). *Human evolution, language and mind: A psychological and archaeological inquiry*. Cambridge University Press.

[CR74] Norenzayan, A., Shariff, A. F., Gervais, W. M., Willard, A. K., McNamara, R. A., Slingerland, E., & Henrich, J. (2016). The cultural evolution of prosocial religions. *Behavioral and Brain Sciences*. 10.1017/S0140525X14001356 10.1017/S0140525X14001356 26785995

[CR75] Northoff, G., & Stanghellini, G. (2016). How to link brain and experience? Spatiotemporal psychopathology of the lived body. *Frontiers in Human Neuroscience,**10*, 76. 10.3389/fnhum.2016.00172 27199695 10.3389/fnhum.2016.00172 PMC4849214

[CR76] Otto, R. (1923). *The idea of the holy*. Oxford University Press Inc.

[CR77] Park, S., & Baxter, T. (2022). Schizophrenia in the flesh: Revisiting schizophrenia as a disorder of the bodily self. *Schizophrenia Research,**242*, 113–117. 10.1016/j.schres.2021.12.031 34996674 10.1016/j.schres.2021.12.031

[CR78] Parnas, J., & Henriksen, M. G. (2014). Disordered self in the schizophrenia spectrum: A clinical and research perspective. *Harvard Review of Psychiatry,**22*(5), 251–265. 10.1097/HRP.0000000000000040 25126763 10.1097/HRP.0000000000000040 PMC4219858

[CR79] Parnas, J., Urfer-Parnas, A., & Stephensen, H. (2021). Double bookkeeping and schizophrenia spectrum: Divided unified phenomenal consciousness. *European Archives of Psychiatry and Clinical Neuroscience,**271*(8), 1513–1523. 10.1007/s00406-020-01185-0 32901298 10.1007/s00406-020-01185-0 PMC8563555

[CR80] Peirce, C. S. (1991). *Peirce on signs*. University of North Carolina Press.

[CR81] Poletti, M., & Raballo, A. (2022). (Developmental) motor signs: Reconceptualizing a potential transdiagnostic marker of psychopathological vulnerability. *Schizophrenia Bulletin,**48*(4), 763–765. 10.1093/schbul/sbac026 35265980 10.1093/schbul/sbac026 PMC9212093

[CR82] Powers, A. R., Hillock, A. R., & Wallace, M. T. (2009). Perceptual training narrows the temporal window of multisensory binding. *The Journal of Neuroscience: the Official Journal of the Society for Neuroscience,**29*(39), 12265–12274. 10.1523/JNEUROSCI.3501-09.2009 19793985 10.1523/JNEUROSCI.3501-09.2009 PMC2771316

[CR83] Pulvermüller, F. (2005). Brain mechanisms linking language and action. *Nature Reviews. Neuroscience,**6*(7), 576–582. 10.1038/nrn1706 15959465 10.1038/nrn1706

[CR84] Rapoport, J., Giedd, J., & Gogtay, N. (2012). Neurodevelopmental model of schizophrenia: Update 2012. *Molecular Psychiatry,**17*(12), 1228–1238. 10.1038/mp.2012.23 22488257 10.1038/mp.2012.23 PMC3504171

[CR85] Rappaport, R. A. (1999). *Ritual and religion in the making of humanity*. Cambridge University Press.

[CR86] Reddish, P., Ronald, F., & Bulbulia, J. (2013). Let’s dance together: Synchrony, shared intentionality and cooperation. *PLoS ONE,**8*(8), e71182. 10.1371/journal.pone.0071182 23951106 10.1371/journal.pone.0071182 PMC3737148

[CR87] Renfrew, C., Frith, C., & Malafouris, L. (2008a). Introduction. The sapient mind: Archaeology meets neuroscience. *Philosophical Transactions of the Royal Society of London. Series b, Biological Sciences,**363*(1499), 1935–1938. 10.1098/rstb.2008.0016 18292055 10.1098/rstb.2008.0016 PMC2394570

[CR88] Renfrew, C., Frith, C., Malafouris, L., & Frey, S. H. (2008b). Tool use, communicative gesture and cerebral asymmetries in the modern human brain. *Philosophical Transactions of the Royal Society b: Biological Sciences,**363*(1499), 1951–1957. 10.1098/rstb.2008.0008 10.1098/rstb.2008.0008 PMC2606701 18292060

[CR89] Rietveld, E. (2014). A rich landscape of affordances. *Ecological Psychology,**26*, 325–352. 10.1080/10407413.2014.958035

[CR90] Robb, J. E. (1998). The archaeology of symbols. *Annual Review of Anthropology,**27*, 329–346. 10.1146/annurev.anthro.27.1.329

[CR91] Sass, L. (1992a). *Madness and modernism: Insanity in the light of modern art, literature, and thought*. Harvard University Press.

[CR92] Sass, L. A. (1992b). Heidegger, schizophrenia and the ontological difference. *Philosophical Psychology,**5*(2), 109–132. 10.1080/09515089208573047

[CR93] Schiffman, J., Mittal, V., Kline, E., Mortensen, E. L., Michelsen, N., Ekstrøm, M., Millman, Z. B., Mednick, S. A., & Sørensen, H. J. (2015). Childhood dyspraxia predicts adult-onset nonaffective-psychosis-spectrum disorder. *Development and Psychopathology,**27*(4), 1323–1330. 10.1017/S0954579414001436 26439077 10.1017/S0954579414001436 PMC12224641

[CR94] Schneider, K. (2007). *Klinische Psychopathologie*. Georg Thieme Verlag.

[CR96] Škodlar, B., & Henriksen, M. G. (2019). Toward a phenomenological psychotherapy for schizophrenia. *Psychopathology,**52*(2), 117–125. 10.1159/000500163 31163426 10.1159/000500163

[CR97] Sosis, R. (2000). Religion and intragroup cooperation: preliminary results of a comparative analysis of utopian communities. *Cross-Cultural Research,**34*, 70–87. 10.1177/106939710003400105

[CR98] Stanghellini, G., Aragona, M., Gilardi, L., & Ritunnano, R. (2022). The person’s position-taking in the shaping of schizophrenic phenomena. *Philosophical Psychology,**36*(7), 1261–1286. 10.1080/09515089.2022.2144192

[CR99] Stevenson, R. A., Ghose, D., Fister, J. K., Sarko, D. K., Altieri, N. A., Nidiffer, A. R., Kurela, L. R., Siemann, J. K., James, T. W., & Wallace, M. T. (2014). Identifying and quantifying multisensory integration: A tutorial review. *Brain Topography,**27*(6), 707–730. 10.1007/s10548014-0365-7 24722880 10.1007/s10548-014-0365-7

[CR100] Storch, A. (1924). *The primitive archaic forms of inner experiences and thought in schizophrenia: A genetic and clinical study of schizophrenia*. Nervous and Mental Disease Publishing Company.

[CR101] Stout, D., & Hecht, E. E. (2017). Evolutionary neuroscience of cumulative culture. *Proceedings of the National Academy of Sciences,**114*(30), 7861–7868. 10.1073/pnas.1620738114 10.1073/pnas.1620738114 PMC5544267 28739892

[CR102] Stout, D., Toth, N., Schick, K., & Chaminade, T. (2008). Neural correlates of early stone age toolmaking: Technology, language and cognition in human evolution. *Philosophical Transactions of the Royal Society b: Biological Sciences,**363*(1499), 1939–1949. 10.1098/rstb.2008.0001 10.1098/rstb.2008.0001 PMC2606694 18292067

[CR103] Tattersall, I. (2016). A tentative framework for the acquisition of language and modern human cognition. *Journal of Anthropological Sciences,**94*, 157–166. 10.4436/JASS.94030 27014833 10.4436/JASS.94030

[CR104] Tattersall, I. (2017). How can we detect when language emerged? *Psychonomic Bulletin & Review,**24*(1), 64–67. 10.3758/s13423-016-1075-9 27368620 10.3758/s13423-016-1075-9

[CR105] Tonna, M., Lucarini, V., Borrelli, D. F., Parmigiani, S., & Marchesi, C. (2023a). Disembodiment and language in schizophrenia: An integrated psychopathological and evolutionary perspective. *Schizophrenia Bulletin,**49*(1), 161–171. 10.1093/schbul/sbac146 36264669 10.1093/schbul/sbac146 PMC9810023

[CR106] Tonna, M., Lucarini, V., Lucchese, J., Presta, V., Paraboschi, F., Marsella, F., Daniel, B. D., Vitale, M., Marchesi, C., & Gobbi, G. (2023b). Posture, gait and self-disorders: An empirical study in individuals with schizophrenia. *Early Intervention in Psychiatry,**17*(5), 447–461. 10.1111/eip.13340 37156494 10.1111/eip.13340

[CR107] Tonna, M., Marchesi, C., & Parmigiani, S. (2019). The biological origins of rituals: An interdisciplinary perspective. *Neuroscience & Biobehavioral Reviews,**98*, 95–106. 10.1016/j.neubiorev.2018.12.031 30610910 10.1016/j.neubiorev.2018.12.031

[CR108] Tonna, M., Ponzi, D., Palanza, P., Marchesi, C., & Parmigiani, S. (2020). Proximate and ultimate causes of ritual behavior. *Behavioural Brain Research.,**393*, 112772. 10.1016/j.bbr.2020.112772 32544508 10.1016/j.bbr.2020.112772

[CR109] Toro, R., Konyukh, M., Delorme, R., Leblond, C., Chaste, P., Fauchereau, F., Coleman, M., Leboyer, M., Gillberg, C., & Bourgeron, T. (2010). Key role for gene dosage and synaptic homeostasis in autism spectrum disorders. *Trends in Genetics,**26*(8), 363–372. 10.1016/j.tig.2010.05.007 20609491 10.1016/j.tig.2010.05.007

[CR110] Tripp, A. J., Cook, A., & von Petzinger, G. (2014). *Encyclopedia of global archaeology*. Springer.

[CR111] Walther, S., & Strik, W. (2012). Motor symptoms and schizophrenia. *Neuropsychobiology,**66*(2), 77–92. 10.1159/000339456 22814247 10.1159/000339456

[CR112] Watkins, T. (2005). The Neolithic revolution and the emergence of humanity: A cognitive approach to the first comprehensive world-view. In J. Clarke (Ed.), *Archaeological perspectives on the transmission and transformation of culture in the eastern Mediterranean. *Council for British Research in the Levant.

[CR113] White, R. (1992). Beyond art: Toward an understanding of the origins of material representation in Europe. *Annual Review of Anthropology,**21*(1), 537–564. 10.1146/annurev.an.21.100192.002541

[CR114] Wyrsch, J. (1949). *Die person des Schizophrenen*. Haupt Verlag.

